# Assessment of the therapeutic value of new medicines marketed in Australia

**DOI:** 10.1186/2052-3211-6-2

**Published:** 2013-06-13

**Authors:** Agnes I Vitry, Ng Huah Shin, Pauline Vitre

**Affiliations:** 1 Quality Use of Medicines and Pharmacy Research Centre, Sansom Institute for Health Sciences, School of Pharmacy and Medical Sciences, University of South Australia, Adelaide, Australia

**Keywords:** Medicine regulation, New medicine, Therapeutic value, Innovation

## Abstract

**Background:**

The belief that all new medicines bring a therapeutic innovation and better health outcomes is widely shared among the public, health professionals and policy makers.

**Objectives:**

To examine the therapeutic value of new medicines marketed in Australia using two classification systems.

**Methods:**

The therapeutic value of new medicines was categorised using the Motola’s s and the Ahlqvist-Rastad’s systems for all approvals made by the Australian Therapeutics Goods Administration (TGA) between 2005 and 2007. Scores were assigned independently by the three authors on the basis of the Public Summary Documents and *Prescrire*’ review articles.

**Results:**

Overall, 217 approval recommendations were made including 81 (37.3%) for new indications and 69 (31.8%) for new medicines. In Motola’s rating system, 31 (52.5%) of the 59 drugs were rated as pharmacological or technological innovations and 28 (47.5%) were rated as therapeutic innovations. Only seven of the 59 drugs (11.9%) were rated as important innovations. In Ahlqvist-Rastad’s system, only a third of the new drugs were rated as “added therapeutic value”.

**Conclusion:**

Only a minority of the new medicines marketed in Australia provide added therapeutic value compared to existing treatments. Stricter regulatory approval criteria would ensure better safety of the public and simplify the reimbursement processes.

## Introduction

The definition of what constitutes an innovation varies depending on the perspective adopted. From an economic perspective, pharmaceutical products are considered innovative as long as they are new and the success of innovation is defined in terms of sales, with the assumption that higher sales is a measure of the intrinsic worth of the innovation [1]. Patents are delivered to new medicines to protect any kind of innovations such as a novel chemical structure or a new formulation even if it does not translate into a health benefit [2]. From a public health perspective, the value of new medicines lies in their “therapeutic” value and the health benefits that they can generate for patients as well as for the society such as years of life saved, better quality of life or better tolerance [1].

A few studies have assessed the therapeutic innovation of new medicines. In Canada, 68 (5.9%) of the 1147 newly appraised drugs between 1990 and 2003 were found to be “breakthrough” drugs [3]. In France, the French independent medical journal, *Prescrire* has assessed the therapeutic value of new drugs marketed in France for almost 30 years. Between 2000 and 2009, of the 984 new medicines or new indications approved in France, more than half did not provide anything new [4]. At the European level, only a minority of the 88 biotechnological (25%) and 163 non-biotechnological products (29%) approved by the European Medicines Agency (EMA) between 1995 to 2004 were categorised as innovative [5].

However, the belief that all new medicines bring a therapeutic innovation and better health outcomes is widely shared among the public, health professionals and policy makers [6]. The recognition of the innovation provided by new medicines was an important issue considered during the negotiations of the Australia-United States (US) Free Trade Agreement (AUSFTA) in 2004 [7,8]. US negotiators were concerned that the pricing policy administered by the Pharmaceutical Benefits Scheme (PBS) in Australia failed to adequately reward pharmaceutical innovations [8,9].

The valuation of therapeutic innovation has been mainly addressed in the context of medicines reimbursement systems but is not a factor taken into consideration by medicine regulatory agencies. However, classification systems to categorise the therapeutic value of new medicines have been developed by some medicine agencies [10] and medical journals including *Prescrire* in France [4]. Such classification systems can help informing health professionals and the public about the real therapeutic value of new medicines. They can also be used to evaluate the new medicines over time and across therapeutic categories and to assess the impact of medicine policies and regulations [3]. The objective of this study was to examine the therapeutic value of new medicines marketed in Australia using two classification systems previously developed.

## Methods

The therapeutic value of new medicines was categorised using two classification systems, the Motola’s system [5] and the Ahlqvist-Rastad’s system [11]. The Motola’s system is currently used by the Italian Medicines Agency for ranking innovation of new drugs [10]. The degree of therapeutic innovation is scored by evaluating the seriousness of the disease, the availability of previous treatments, and the extent of the therapeutic effect, according to an algorithm (Table 1) [5]. The medicines are finally categorised into one of three degrees of therapeutic innovation (important, moderate or modest) or a pharmacological (new mechanism of action) or technological (new chemical or biotechnological) innovation without evidence for better efficacy, safety or kinetics than existing treatments. The Ahlqvist-Rastad’s system was proposed by *Prescrire* and the Swedish Medical Products Agency to classify new medicines into five main categories with additional subcategories (Table 2) [11].

**Table 1:** Therapeutic value of new drugs assessed with the Motola’s rating system

**Disease seriousness**				
**Therapeutic innovation**	**Drug for serious diseases**	**Drug for risk factors for serious diseases**	**Drug for non serious diseases**	**Total**
Important	7	-	-	7 (11.9%)
Moderate	17	-	-	17 (28.8%)
Modest	3	1	-	4 (6.8%)
Pharmacological	5	1	-	6 (10.2%)
Technological	22	1	2	25 (42.4%)
TOTAL	54 (91.5%)	3 (5.1%)	2 (3.4%)	59 (100.0%)

**Table 2:** Therapeutic value of new drugs assessed with Ahlqvist-Rastad’s rating system

**Ahlqvist-Rastad’s rating system**			**N**	**%**
Drug for conditions with no currently available treatment	A		0	0.0
Added therapeutic value*	B	B1	18	30.5
		B4	1	1.7
		Subtotal	19	32.2
Similar therapeutic value**	C	C1	5	8.5
		C2	20	33.9
		Subtotal	25	42.4
Inferior therapeutic value***	D	D1	2	3.4
		D2	3	5.1
		Subtotal	5	8.5
Uncertain therapeutic value****	E		10	16.9
TOTAL			59	100.0

*The effect (B1)/ safety (B2)/ dosage (B3)/ route of administration (B4) seems to be better for patients than that of previously licensed alternatives.

** First medicine of a new class of agents with similar therapeutic value to that of previously licensed alternatives (C1).

New agent of an existing class with similar therapeutic value to that of previously licensed alternatives (C2).

*** First medicine of a new class with inferior therapeutic value to that of previously licensed alternatives (D1).

New agent of an existing class with inferior therapeutic value to that of previously licensed alternatives (D2).

****New agent whose therapeutic value remains unknown because evaluation is limited to its effects on surrogate end points.

All recommendations made by the Australian Drug Evaluation Committee (ADEC) between 2005 and 2007 were accessed from the Therapeutics Goods Administration (TGA) website (http://www.tga.gov.au/archive/committees-adec-resolutions.htm) and classified according to type of approvals and therapeutic class using the Anatomic Therapeutic Chemical (ATC) classification system. Vaccines, diagnostics and desensitising agents were excluded from the analysis. When new medicines had more than one indication, therapeutic scores were assigned to each indication.

The Motola and Ahlqvist-Rastad’s rating scores were assigned independently by the three authors for all indications. The sources of information used to inform the assessment were (1) the Public Summary Documents (PSDs) summarizing the Australian Pharmaceutical Benefits Advisory Committee (PBAC) assessments of all applications for funding of new medicines under the Pharmaceutical Benefits Scheme (PBS) available till November 2007; (2) the reviews published by *Prescrire* till April 2008. These sources were chosen as they are independent and provide high quality comprehensive reviews of the clinical evidence for new medicines. In the few cases where information was not available from one of these two sources, the Australian Product Information and the European Public Assessment Report (EPAR), which summarizes the reasons for approval of new drugs by the European Medicines Agency (EMA), were used. Disagreements between the 3 authors in assessing medicines were resolved by consensus meetings. Descriptive comparisons were made between ratings obtained by the Motola’s system and Ahlqvist-Rastad’s systems. An analysis of the possible reasons for the differences in ratings was also undertaken on the basis of the information provided in the PDSs and *Prescrire*’s articles.

## Results

**Figure 1:**
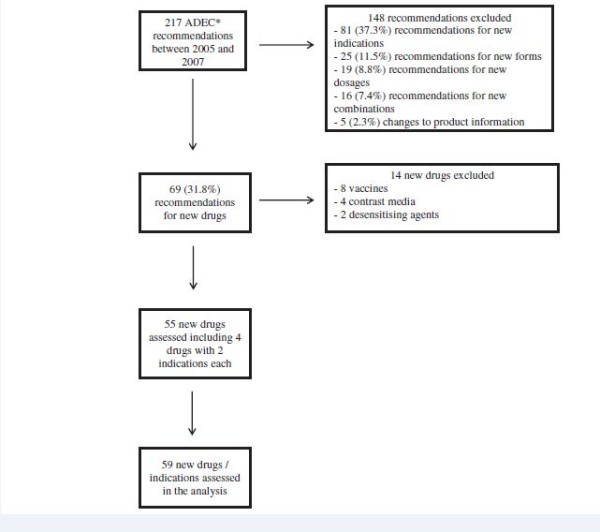
**Flow chart of the new drugs / indications included in the analysis.** * ADEC: Australian Drug Evaluation Committee.

Figure 1 describes the flow chart of the new drugs / indications included in the analysis.

Between 2005 and 2007, 217 recommendations were made by ADEC including 81 (37.3%) recommendations for new indications, 69 (31.8%) for new drugs. The remaining was for new forms (11.5%), new dosages (8.8%), new combinations (7.4%), changes to Product Information (2.3%) and generic products (0.9%). The number of changes to Product Information and approvals of generics were low as most changes to Product Information and most approvals of generics do not need to be reviewed by ADEC. Anti-infectives represented 27.5% of the new drugs, antineoplastics and immunomoduling agents 18.8%, drugs for diseases of the alimentary tract and metabolism 8.7%, drugs for the nervous system 8.7%, drugs for the cardiovascular system 4.8% and drugs for blood disorders 4.8%.

The assessment of the therapeutic value included 55 out of the 69 new drugs approved by the ADEC. Fourteen drugs were excluded from the analysis, including eight vaccines, four contrast media and two desensitising agents. As there were four new drugs (dasatinib, posaconazole, pregabalin and sunitinib) with two different indications, a total of 59 new drugs/indications were rated for their therapeutic value.

For 46 (78%) drugs/indications, either the *Prescrire*’s article or the Public Summary Documents were available. In five (8%) cases, the European Product Assessment Reports (EPAR) was used as there was no *Prescrire*’s review or Public Summary Documents available. In eight (14%) cases, no information could be retrieved from any of these three sources but all these products were biological products (e.g. factor eight inhibitor bypassing fraction, human normal immunoglobulin) except one product, butoconazole. The Product Information was used to rate the therapeutic value of butoconazole.

Most of the new drugs/indications (91.5%) were indicated for serious diseases such as HIV, hepatitis, cancer, epilepsy and invasive fungal infections (Table 1). Three (5.1%) were indicated for the management of risk factors for serious diseases such as nicotine dependence, osteoporosis and hypertension and two (3.4%) for the treatment of non-serious disease such as vulvo-vaginal candidiasis and urinary incontinence.

In the Motola's system, 31 (52.5%) of the 59 drugs were rated as pharmacological or technological innovations and 28 (47.5%) were rated as therapeutic innovations (Table 1). A minority (11.9%) were rated as important innovation. Most of the biological products were rated as technological innovation with therapeutic roles similar to the existing ones.

In the Ahlqvist-Rastad’s system, no drug was approved for “conditions with no currently available treatment” (Table 2). Nineteen (32.2%) new drugs were rated as “added therapeutic value” and 25 (42.4%) as “similar therapeutic value”. Five (8.5%) were rated as “inferior therapeutic value” and ten (16.9%) as “uncertain therapeutic value”.

The comparison between the scores obtained with Motola’s rating system and the Ahlqvist-Rastad’s rating system showed that all drugs rated as “added therapeutic value” with the Ahlqvist-Rastad’s system were graded as important (36.8%), moderate (47.4%) or modest (15.8%) innovations with Motola’s rating system (Table 3). Most of the drugs (88.0%) rated as “similar therapeutic value” and all drugs classified as “inferior therapeutic value” were graded as pharmacological or technological innovations. Three drugs (abatacept, fulvestrant and pregabalin) were rated as “similar therapeutic value” in the Ahlqvist-Rastad’s system and rated as moderate innovation in the Motola’s system. The reason is that none of these three medicines had been shown to have superior efficacy compared to standard treatments. However, they could provide another therapeutic option for conditions where there were only limited treatments.

**Table 3:** Comparison between scores obtained with the Motola’s rating system and the Ahlqvist-Rastad’s rating system

	**Ahlqvist-Rastad’s rating system**				
**Motola’s rating system**	**Added therapeutic value**	**Similar therapeutic value**	**Inferior therapeutic value**	**Uncertain therapeutic value**	**Total**
Important	7 (36.8%)	-	-	-	7
Moderate	9 (47.4%)	3 (12.0%) *	-	5 (50.0%) **	17
Modest	3 (15.8%)	-	-	1 (10.0%) ***	4
Pharmacological	-	2 (8.0%)	2 (40.0%)	2 (20.0%)	6
Technological	-	20 (80.0%)	3 (60.0%)	2 (20.0%)	25
TOTAL	19	25	5	10	59

* Abatacept, fulvestrant and pregabalin.

** Maraviroc, natalizumab, nitric oxide, pegvisomant and sunitinib.

*** Palifermin.

Six drugs (maraviroc, natalizumab, nitric oxide, pegvisomant, sunitinib and palifermin) were rated as “uncertain therapeutic value” in the Ahlqvist-Rastad’s system and rated as therapeutic innovations in the Motola’s system. The reasons for the “uncertain” ratings were either because of the quality of scientific evidence (absence of a direct comparison with the reference treatment for natalizumab, use of surrogate outcomes for maraviroc, maturity of data for sunitinib) or because of safety concerns (risk of progressive multifocal leukoencephalopathy with natalizumab).

## Discussion

The assessment of the therapeutic value of new medicines approved in Australia between 2005 and 2007 included a total of 59 new drugs/indications. In Motola’s rating system, 31 (52.5%) of the 59 drugs were rated as pharmacological or technological innovations and 28 (47.5%) were rated as therapeutic innovations. Only a minority (11.9%) were rated as important innovations. In Ahlqvist-Rastad’s system, only a third of new drugs were rated as “added therapeutic value”.

Our results are similar to those found in other countries. In Europe, of 176 medicines approved by EMA between 1995 and 2004, 49% of the new drugs were pharmacological or technological innovations and 51% were therapeutic innovations [5]. Of the 122 medicines approved by EMA between 1999 and 2005, only 13 (10%) were shown to be superior to already available medicines in terms of a statistically significant difference in primary clinical endpoints [12].

Classification using both systems was broadly consistent. The Motola’s system did not include categories for inferior therapeutic value and uncertain benefits. The Ahlqvist-Rastad’s system did not include a category for medicines that could have better efficacy but increased risk of adverse effects. A classification system that includes all these elements would be really valuable for providing an informative summary of the clinical significance of new medicines. This may contribute to educate the public about the real therapeutic value of new medicines and change the beliefs that all new medicines bring a therapeutic innovation.

The evaluation of the therapeutic value of new medicines in both systems was complicated by significant scientific uncertainties. In our study, 17% of the new drugs/indications were rated as of uncertain therapeutic value. A study found that more than 40% of all submissions by the Common Drug Review in Canada and the PBAC in Australia were associated with considerable clinical uncertainty [13]. Significant uncertainty around comparative clinical efficacy caused by the use of inappropriate study design, comparators and surrogate end points, was identified as a key issue in coverage decisions across three national jurisdictions in Britain, Australia and Canada. The threshold of acceptable uncertainty is often considered to be higher for new medicines targeting life-threatening diseases. However, even in this situation, accepting a high level of uncertainty means that some patients will be exposed to a high risk of severe adverse effects without gaining any benefit.

The current approval regulations do not require the demonstration of any improvement in terms of efficacy or safety for new medicines compared to existing products. This means that, in practice, medicines with an uncertain or lower benefit-to-risk ratio can be given the “benefit of doubt” and be marketed worldwide [14]. Calls have be made for a strengthening of the criteria for marketing approval including the requirement to demonstrate at least a minimal therapeutic improvement, in particular for cancer drugs [15,16], and requirement of active-controlled trials [17,18]. Making therapeutic value the criterion for marketing authorization may become a realistic option as the role of third party payers has become more prominent, including in Australia where “time-to-market no longer means time-to-licensing but time-to-reimbursement” [18]. A better integration of the functions of regulatory agencies and health technology assessment bodies would facilitate the assessment of the therapeutic value of new medicines and improve the health of the public [17,19].

A limitation of our study is that we assessed the therapeutic value of drugs based on data available at the time of marketing authorisation. However, new evidence may become available after the marketing approval, either as new evidence of efficacy compared to alternative treatments or new evidence of efficacy on clinical outcomes rather than on surrogate outcomes or reporting of new serious adverse effects. This new evidence may change the assessment of the therapeutic value of new drugs, in either a better or worse direction [20]. A second limitation of this study is that it is based on publicly available evidence. At the time of our study, the Therapeutics Goods Administration was not publishing any evaluation reports on newly approved medicines. Since October 2009, the TGA has been publishing the AusPARs, comprehensive reports that provide information about the evaluation of new medicines and the considerations that led the TGA to approve or not approve an application. However, these reports are long and complex and do not provide an informative rating of the therapeutic value of new medicines. Another limitation of this study is the lack of gold standard methodology for the evaluation of the therapeutic value of new medicines. Regulatory agencies do no currently use standardised processes but mostly rely on expert judgment. A number of quantitative and semi-quantitative methods designed to weigh all the relevant efficacy and safety data have been proposed to make more objective, transparent and consistent decisions [21]. However, their validity remains to be assessed in the regulatory context. In our study, we have used independent and internationally recognised sources of reviews on new medicines to inform the value rating in both the Motola’s system and Ahlqvist-Rastad’s system.

## Conclusion

Use of a simple categorisation system could provide a useful, simple and transparent way to better inform the public and health professionals of the therapeutic value of new medicines. Currently, only a minority of the new medicines marketed in Australia provide added therapeutic value compared to existing treatments. By strengthening approval criteria for new medicines, the Australian government could ensure a better safety of the public and streamline registration and reimbursement processes.
